# Green, Sustainable Synthesis of γ-Fe_2_O_3_/MWCNT/Ag Nano-Composites Using the *Viscum album* Leaf Extract and Waste Car Tire for Removal of Sulfamethazine and Bacteria from Wastewater Streams

**DOI:** 10.3390/nano12162798

**Published:** 2022-08-15

**Authors:** Mansooreh Khalatbary, Mohammad Hossein Sayadi, Mahmood Hajiani, Mohsen Nowrouzi, Shahin Homaeigohar

**Affiliations:** 1 Department of Environmental Engineering, Faculty of Natural Resources and Environment, University of Birjand, Birjand P.O. Box 97175/615, Iran; 2 Department of Science and Biotechnology, Faculty of Nano and Bio Science and Technology, Persian Gulf University, Bushehr 75169-13798, Iran; 3 School of Science and Engineering, University of Dundee, Dundee DD1 4HN, UK

**Keywords:** γ-Fe_2_O_3_/MWCNTs/Ag nanocomposite, sulfamethazine, antibacterial activity, Taguchi design

## Abstract

Multi-walled carbon nanotubes (MWCNTs) decorated with Ag nanoparticles (NPs) are bifunctional adsorbent nanomaterials with antibacterial activity. They can be magnetically recovered from wastewater in case of coupling with γ-Fe_2_O_3_. In this study, for the first time, an environmentally friendly technique was applied to prepare a nanocomposite (NC) material composed of γ-Fe_2_O_3_/MWCNT/Ag by using Bridgestone disposable tires and *Viscum album* leaves extract. γ-Fe_2_O_3_/MWCNTs/Ag NC was employed for the removal of sulfamethazine (SMT) from aqueous solutions. Under the optimized conditions determined via the Taguchi method, the highest SMT adsorption capacity of the γ-Fe_2_O_3_/MWCNT/Ag NC was measured to be 47.6 mg/g. The experimental data fitted well with the pseudo-second-order kinetic model and the Langmuir isotherm. The thermodynamic parameters implied that the adsorption process was endothermic. In addition to adsorption of the drug pollutant, the NC demonstrated a superior antibacterial activity against Gram-positive bacteria. The reusability test also showed that over 79% SMT can be removed using γ-Fe_2_O_3_/MWCNTs/Ag NC even after four adsorption cycles. Taken together, γ-Fe_2_O_3_/MWCNTs/Ag NC was proven to be a promising antibacterial nano-adsorbent for wastewater treatment.

## 1. Introduction

In the recent years, drug compounds have been regarded as one of the most concerning water contaminants due to their variety, wide utilization, and stability in the aqueous environment. These compounds are a subgroup of micro-pollutants that enter the environment and water streams through the disposal of wastewater or agricultural run-off. Among the pharmaceutical compounds, antibiotics have received great attention due to their capability to induce antibiotic resistance in the pathogenic bacteria [1]. Sulfamethazine (SMT) is a sulfonamide antibiotic that is widely used in the treatment of bacterial infections, such as a sore throat, sinusitis, bronchitis, prostatitis, and urinary tract infections [2]. Among the pharmaceutical materials, this compound is minimally (10–20%) metabolized and its major fraction is removed in urine and feces [3]. The existence of SMT (with minor concentrations of ng/L to μg/L) has been detected in water and wastewater streams worldwide [4]. This pollutant, even at such insignificant concentrations, can enter the food cycle and drive drug resistance, thus posing many environmental risks. In recent years, various methods have been applied to remove such drug contaminants including coagulation and flocculation, flotation with soluble air, filtration, biological degradation, optical decomposition, and advanced oxidation process [5,6,7,8]. Some of these techniques are challenging due to their high operation costs and time-inefficiency. In contrast, adsorption is a low cost, effective technique that can be performed without the generation of toxic by-products [9,10]. The effectiveness of this method depends on the physicochemical properties of the adsorbent, including porosity, specific surface area, surface polarity, and hydrophobicity [11]. Nowadays, activated carbon is a golden benchmark for adsorption due to its promising chemical properties, high specific surface area, and pore size [12]. Of the same family, carbon nanofibers and CNTs can also be regarded as next generation adsorbents, particularly for the removal of hazardous organic and drug pollutants [13,14,15].

The widespread manufacture of tires worldwide and technical difficulty of their recycling have inspired researchers to pyrolyze rubber for the production of carbon materials (containing up to 93 wt.% carbon) [11]. This carbon remnant can be employed as a precursor for the synthesis of carbon nanomaterials such as CNTs [16]. Despite the optimum adsorption performance of CNTs, their separation from aqueous solutions is notably difficult. To resolve this issue, one established strategy is coupling the CNTs with magnetic NPs, thereby readily separating the assembly from the treated aqueous solution using a magnetic field [17]. In this regard, Ahamad et al. [18] coupled amino-functionalized MWCNTs with Fe_3_O_4_ for the removal of methylene blue dye from water. The presence of the magnetic phase facilitated the facile and prompt separation of the composite particles from the aqueous solution even in large volumes. Among the studied magnetic materials, maghemite (γ-Fe_2_O_3_) has been proven to offer superior magnetic properties, reactivity, and biocompatibility. Benefitting from the optimum adsorption capacity and magnetic property of MWCNT and maghemite, respectively, very recently, Khalatbari et al. [16] demonstrated that a composite nanosystem comprising γ-Fe_2_O_3_, MWCNT, and cellulose can potentially act as a proper nano-adsorbent for the separation of malachite green dye from water. 

CNTs can also achieve enhanced surface reactivity by surface decoration with metal NPs. As a result of this strategy, the electrical conductivity, catalysis, antimicrobial activity, and chemical stability of CNTs can be improved [19,20]. Metal NPs made of lead (Pb), silver (Ag), gold (Au), nickel (Ni), platinum (Pt), and copper (Cu) have been mainly used for the above mentioned purposes [21]. Among these types of NPs, Ag NPs have been proven to be effective in the elimination of organic and inorganic pollutants due to their excellent specific surface area [22] and outstanding antibacterial and environmentally friendly properties [23]. Silver is also appropriate for various industrial applications as a catalyst due to its cost-effectiveness and availability [24]. The hybridization of CNTs with Ag NPs results in an optimized antibacterial activity. Despite the merits that Ag NPs offer, their synthesis methods are by no means environmentally friendly [25]. This research aspiration, i.e., green synthesis of Ag NPs, has been seriously pursued by researchers across the world. In this regard, plant extracts have been mainly investigated owing to their negligible toxicity, low cost, and the possibility of fine tuning the final structure/properties of the Ag NPs [26]. Plant extracts contain polyphenolic compounds and flavonoids, which can reduce the Ag cation to the zero-valent Ag particles and minimize the agglomeration of the biosynthesized NPs [27].

In this study, for the first time, to the best of our knowledge, the extract of *Viscum album* leaves was employed for the biosynthesis of Ag NPs. The rationale behind the use of this plant-derived compound is its low cost and large availability in our local area, as well as its unique chemical properties, whereby acting as both a reducing and capping agent [26,27]. The biosynthesized Ag NPs were subsequently hybridized with γ-Fe_2_O_3_/MWCNTs obtained from a Bridgestone disposable tire derived carbon precursor. γ-Fe_2_O_3_/MWCNTs/Ag NCs were employed for the removal of SMT from wastewater streams and for the inactivation of two different bacteria strains, *Escherichia coli (E. Coli)* and *Staphylococcus aureus* (*S. aureus*). To optimize the experimental conditions of the SMT adsorption process including pH, the adsorbent dosage, initial SMT concentration, contact time, temperature, and bed height, the Taguchi design was applied.

## 2. Materials and Methods

### 2.1. Chemicals and Reagents

Dimethylformamide (DMF), ammonia (NH_3_), epichlorohydrin (C_3_H_5_ClO), iron chloride tetrahydrate (FeCl_2_.4H_2_O), silver nitrate (AgNO_3_, 99.9%), sodium hydroxide (NaOH, 99%), hexamethylenetetramine ((CH_2_)_6_N_4_), hydrochloric acid (99%, HCl), sodium nitrate (NaNO_3_), carbamoylsulfamic acid (CH_4_N_2_O_4_S), ethanol, sodium borohydride (NaBH_4_), sulfamethazine (4-amino-N-[4,6-Methyl-2-pyrimidinyl] benzenesulfonamide, purity > 99%), zeocin and kanamycin antibiotics were purchased from Sigma Aldrich (Burlington, MA, USA). Distilled water was obtained from the Milli-Q system. For the synthesis of Ag NPs, healthy mistletoe leaves (*Viscum album*) were collected from different gardens in Birjand. The Bridgestone disposable tires were used to synthesize MWCNTs. Ferrocene was purchased from Sigma Aldrich (Burlington, MA, USA), and was used as the Fe catalyst.

### 2.2. Synthesis of γ-Fe_2_O_3_ NPs

A total of 26 g of (CH_2_)_6_N_4_, 6 g of NaNO_3_, and 20 g of FeCl_2_.4H_2_O were dissolved in 500 mL of deionized water to form a black precipitate. The obtained precipitate was continuously oxygen pumped at 60 °C for 6 h and then filtered through a filter paper (Whatman No. 1). In the next step, the precipitate remained on the filter paper was washed thrice with ethanol and deionized water to reduce the amount of anions and organic impurities, and then dried at 70 °C for 48 h. Finally, the resulting sample was ground to make the γ-Fe_2_O_3_ NPs [28].

### 2.3. Green Synthesis of MWCNTs

A waste tire was cut into small pieces that were mounted within an oven with a N_2_ atmosphere (with the flow rate of 2 L/min) at 400 °C for 20 min. The remnant was manually chopped in a pounder and the resulting fine powder was used as the carbon precursor of MWCNTs. A total of 1 g of the fine powder was mixed with 55 mg of ferrocene in a vase container, and then transferred into a quartz tube exposed to N_2_ for 1 h. This compound was stirred (40 rpm) at room temperature and then heated at the ignition temperature of 700 °C for 20 min, followed by cooling down to room temperature [29].

To preserve their structure, the synthesized MWCNTs were refined using strong oxidants, i.e., a mixture of concentrated HNO_3_ and H_2_SO_4_ in a volumetric ratio of 1:3, based on the reaction stoichiometry of Fe and acids. The MWCNTs were dispersed in the acid mixture and vigorously stirred at room temperature for 48 h. Thereafter, MWCNTs were washed thrice with deionized water (pH = 7) and later dried for 12 h at 120 °C.

### 2.4. Synthesis of γ-Fe_2_O_3_/ MWCNTs

To synthesize the γ-Fe_2_O_3_/MWCNTs, 26 g of (CH_2_)_6_N_4_, 20 g of FeCl_2_.4H_2_O, and 7 g of NaNO_3_ were dissolved in 500 mL of deionized water. Afterwards, 0.2 g of MWCNTs was added and the mixture was stirred for 1 h and then sonicated. Subsequently, an aqueous ammonia solution (ratio of 1:1) was added to the mixture and further sonicated. The as-prepared suspension was stabilized for 48 h and the obtained precipitate was filtered, washed several times with distilled water and ethanol, and eventually dried at 80 °C for 8 h [30].

### 2.5. Preparation of Viscum Album Leaf Extract

The *Viscum album* leaves were washed with distilled water and then pulverized (100 g). The powder was boiled within a deionized water bath (1 L) at 70 °C for 1 h. The aqueous extract was cooled down to room temperature, filtered by a filter paper (Whatman No. 1) and stored at 50 °C.

### 2.6. Biosynthesis of Ag NPs

Ag NPs were biosynthesized in the dark and within an ultrasonic bath (PARSONIC 7500S, Tehran, Iran). To do so, 70 mL of the *Viscum album* extract and 15 mL of AgNO_3_ solution (0.05 M) were mixed in a container. The pH of the mixture solution was fixed at pH8 using HCl and NaOH (0.1M). The solution was subsequently sonicated in an ultrasonic bath at 70 °C for 20 min. The reduction of Ag^+^ ions to Ag^0^ NPs was monitored visually with the color shift from yellow to dark brown [31].

### 2.7. Synthesis of the γ-Fe_2_O_3_/MWCNT/Ag NC Particles

**Figure 1:**
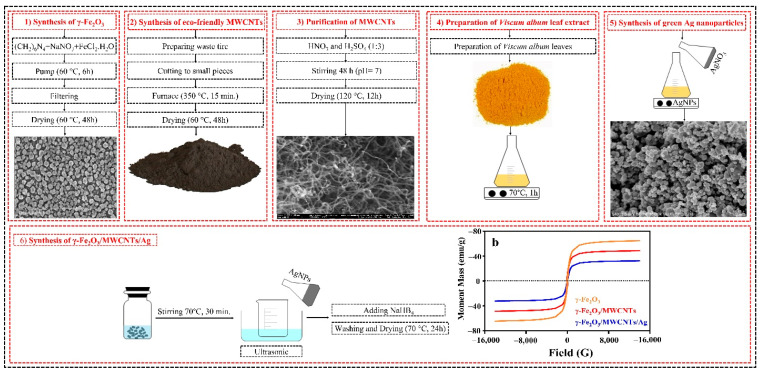
Schematic illustration of the synthesis process of the γ-Fe_2_O_3_/MWCNT/Ag NC particles.

To synthesize the γ-Fe_2_O_3_/MWCNT/Ag NC particles, 2 g of γ-Fe_2_O_3_/MWCNT was dispersed in 50 mL of deionized water and stirred at 80 °C for 30 min. In parallel, 0.06 g of Ag NPs was dispersed in 25 mL of deionized water and sonicated for 15 min. The two suspensions were mixed and heated at 80 °C for 30 min. Thereafter, 0.1 g of NaBH_4_ was dissolved in 25 mL of deionized water and added dropwise to the mixture suspension. After stirring for 30 min, the precipitate was magnetically separated, washed several times with ethanol and deionized water, and eventually dried for 24 h at 60 °C [32]. Figure 1 schematically shows the synthesis procedure of the γ-Fe_2_O_3_/MWCNT/Ag NC particles.

### 2.8. Characterization of the γ-Fe_2_O_3_/MWCNT/Ag NC Particles

The morphology and size of the NC particles were imaged using Field Emission Scanning Electron Microscopy (FESEM) (FESEM-FEI Nanosem 450, Hillsboro, USA), and transmission electron microscopy (TEM, M900). Elemental analysis was performed using an alternating EDS spectrometer (coupled with FESEM). The crystalline structure of the NC particles was analyzed using an X-ray diffractometer (XRD) (MiniFlex 600, Rigaku, Japan) at λ = 0.15418 nm. A vibrating sample magnetometer (VSM) (Lakeshore 7403, OH, USA) was used to determine the magnetic properties of the NC particles. Fourier transforms infrared spectroscopy (Shimadzu, FTIR1650 spectrophotometer, Kyoto, Japan) (using KBr plates) was conducted to characterize the surface chemistry of the NC particles. The size distribution of the NC particles was determined via the Dynamic Light Scattering (DLS) technique using the Zetasizer 3000HS (Malvern, Worcs, UK). X-ray photoelectron spectroscopy (XPS) (Thermofisher Scientific, Waltham, MA, USA) was utilized for chemical analysis. The zeta potential of the NC particles was measured using a zeta potential analyzer (SZ-100z, Horiba Jobin Jyovin, Kyoto, Japan). The thermogravimetric analysis (TGA) was carried out using a Perkin Elmer instrument (TGA8000, Waltham, MA, USA) under N_2_ at the temperature sweep between room temperature and 800 °C. The specific surface area of the NC particles was measured via the Brunauer–Emmett–Teller (BET) technique using a micrometer (Microtrac BEL Corp., Osaka, Japan).

### 2.9. Adsorption Experiments

The γ-Fe_2_O_3_/MWCNT/Ag NC particles were challenged in terms of the SMT removal capacity. In this regard, the effect of NC dosage, pH, SMT initial concentration, contact time, temperature, and bed height on the SMT removal efficiency was investigated. For this purpose, 10 mg of the γ-Fe_2_O_3_/MWCNT/Ag NC particles was added to 100 mL of a SMT aqueous solution (10 mg/L) and the suspension was stirred (at 160 rpm) in a thermostat shaker for 15 min at pH7 and 40 °C. The pH was adjusted by the addition of HCl and NaOH (0.1 M). After the given time intervals, the NC particles were filtered and the SMT concentration was measured with a UV-Vis spectrophotometer at the characteristic wavelength (λ) of 460 nm. The adsorption extent of the NC particles per unit mass (*q_e_*) and the SMT removal efficiency (*RE*%) were calculated via Equations (1) and (2) [33]:

(1) $$q_{e}=\frac{\left(C_{0}-C_{e}\right)V}{m}$$

(2) $$RE\left(\%\right)=\frac{C_{0}-C_{e}}{C_{0}}\times 100\%$$

where *C_0_* and *C_e_* are the initial and equilibrium concentration of SMT (mg/L), respectively. *q_e_* is the amount of the adsorbed SMT at equilibrium and *V* and *m* are the solution volume (L) and the NPs mass (g), respectively.

### 2.10. Design of Experiments by the Taguchi Method

In this study, the Taguchi method (MINITAB software (v. 20)) was employed to determine the optimum operational parameters (pH, SMT initial concentration, adsorbent dosage, time, bed height, and temperature) for the SMT adsorption process at 5 levels (Supplementary Information Table S1). The optimum conditions and significance of each variable were determined based on the signal-to-noise ratio (*S/N*) [34]. The *S/N* ratio in the Taguchi method was estimated using the following Equation (3) [35]:

(3) $$\frac{S}{N}=-10\log\left[\frac{1}{n}\sum_{i=1}^{1}(\frac{1}{y_{i}^{2}}\right)]$$

where *n* is the number of repetitions of an experiment and *y_i_* is the noticed response.

### 2.11. Adsorption Isotherm

The SMT adsorption isotherms were analyzed using the Langmuir and Freundlich models. The Langmuir isotherm model is based on the monolayer, homogeneous adsorption of the adsorbate molecules and is described via Equation (4) [36]:

(4) $$\frac{C_{e}}{q_{e}}=\frac{1}{K_{L}q_{m}}+\frac{C_{e}}{q_{m}}$$

where *q_m_* (mg/g) and *K_L_* (L/mg) are the maximum SMT adsorption amount and the Langmuir coefficient, respectively. In contrast to the Langmuir model, dealing with the adsorption of adsorbate on particular homogeneous sites, the Freundlich isotherm model takes into account an irreversible, multilayer adsorption on a heterogenous surface as described by Equation (5) [37]:

(5) $$lnq_{e}=lnK_{F}+\frac{1}{n_{F}}lnC_{e}$$

where *K_F_* and *n_F_* are the Freundlich constant and heterogeneity factor, respectively.

### 2.12. Adsorption Kinetics

The SMT adsorption kinetics of the NC particles were analyzed using the pseudo first-order Equation (6) and pseudo second-order Equation (7) models [6]:

(6) $$\ln\left(q_{e}-q_{t}\right)=lnq_{e}-K_{1}$$

(7) $$\frac{t}{q_{t}}=\left[\left[\frac{1}{K_{2}q_{e}^{2}}]+[\frac{1}{q_{e}}\right]\right]t$$

where *k*_1_ (min^−1^) and *k*_2_ (min·g/mg) are the rate constants of the pseudo first-order and pseudo second-order adsorption reactions, respectively. *q_e_* and *q_t_* are the SMT adsorption capacity of the NC particles at equilibrium and at time *t* (min), respectively.

### 2.13. Adsorption Thermodynamic

To appraise the effect of temperature on the SMT adsorption process, thermodynamic parameters including standard Gibbs free energy Δ*G*° (kJ/mol), standard enthalpy Δ*H*° (kJ/mol), and standard entropy Δ*S*° (kJ/mol) were determined through the following equations [38]:

(8) ∆G°=∆H°−T∆S°

(9) $$ln\frac{q_{e}}{C_{e}}=\frac{∆H{}^{\circ}}{KT}+\frac{∆S{}^{\circ}}{R}$$

where *T* and *R* are temperature (K) and the gas constant (8.314 J/K·mol), respectively.

### 2.14. Antibacterial Test

The Agar-well diffusion method was applied to characterize the antibacterial activity of γ-Fe_2_O_3_/MWCNTs/Ag NC particles against Gram-negative (*E. coli*) and Gram-positive bacteria (*S. aureus*). To do so, a fresh potato dextrose agar medium was used for the bacterial cultures (10^8^ CFU/mL). Wells (6 mm in diameter) were punched and filled with 50 μL of the agar medium. The control wells were filled with distilled water (negative control) and 7 mL of standard solutions of Zeocin and kanamycin (positive controls). The as-prepared plates were then incubated at 37 °C for 24 h and eventually the antibacterial activity of the NC particles was monitored by measuring the diameter of the inhibition zone.

### 2.15. Data Analyses Using Computer Software

The obtained data were stored in MS Excel software and the Taguchi method was launched via the MINITAB software (v. 20, MINITAB, West Philadelphia, PA, USA).

## 3. Results and Discussion

### 3.1. Structural and Morphological Characteristics of the γ-Fe_2_O_3_/MWCNTs/Ag NC Particles

**Figure 2:**
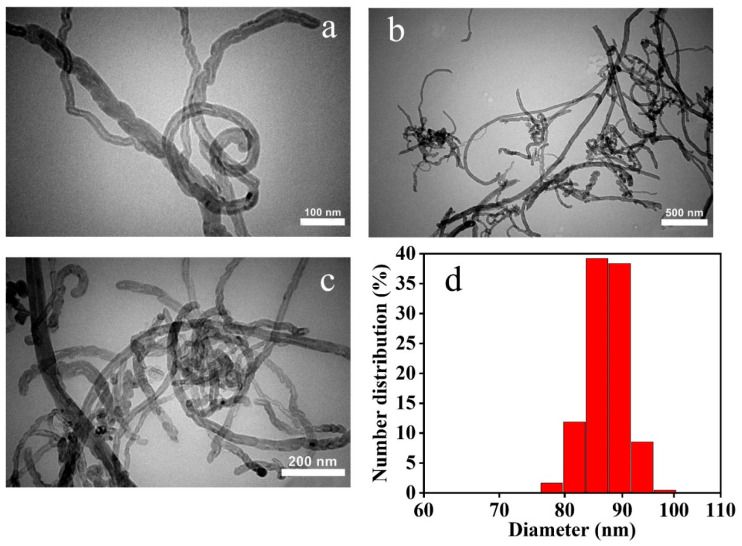
TEM images of MWCNTs (**a**), γ-Fe_2_O_3_/MWCNTs (**b**), and γ-Fe_2_O_3_/MWCNTs/Ag NC particles (**c**). (**d**) Particle size distribution of the γ-Fe_2_O_3_/MWCNTs/Ag NC particles.

As shown in Figure 2a, MWCNTs are in a tubular shape and uniform. Figure 2b,c represent the γ-Fe_2_O_3_/MWCNTs and γ-Fe_2_O_3_/MWCNT/Ag NC particles which firmly hold the γ-Fe_2_O_3_ NPs (homogenously distributed along the MWCNTs), respectively. After the addition of Ag NPs, the number of spherical nanostructures increased, and thus the distinction of the metal and metal oxide NPs becomes indeed challenging. This observation was similarly reported in [16,25]. Figure 2d demonstrates the particle size distribution of the γ-Fe_2_O_3_/MWCNT/Ag NC particles based on the TEM images and DLS analysis. The majority of the NC particles are as small as 75 to 100 nm.

**Figure 3:**
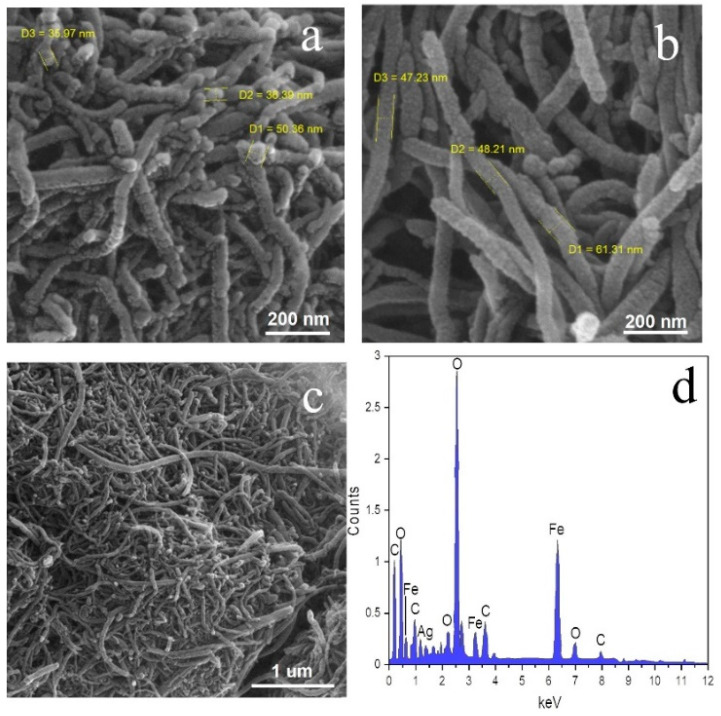
FESEM images of γ-Fe_2_O_3_/MWCNTs (**a**,**b**), and γ-Fe_2_O_3_/MWCNTs/Ag NC (**c**). (**d**) EDX spectrum of γ-Fe_2_O_3_/MWCNTs/Ag NC.

The FESEM images of γ-Fe_2_O_3_/MWCNTs and γ-Fe_2_O_3_/MWCNTs/Ag NC particles are shown in Figure 3a–c. According to these images, MWCNTs are decorated with nodules of γ-Fe_2_O_3_ NPs that are homogenously tethered on their sidewalls. Such a composite structure was similarly reported in [16,21,25]. Evidently, γ-Fe_2_O_3_ or Ag NPs were uniformly spread on the MWCNTs. This finding is further verified by the EDX spectrum, shown in Figure 3d, which reveals the existence of C, O, Ag, and Fe on the surface of the γ-Fe_2_O_3_/MWCNTs/Ag NC particles. Therefore, MWCNTs can properly act as a supporting material and substrate ensuring the uniform dispersion of Ag and γ-Fe_2_O_3_ NPs, as similarly reported by Moazzen et al. [32].

### 3.2. Crystallinity of the γ-Fe_2_O_3_/MWCNT/Ag NC Particles

**Figure 4:**
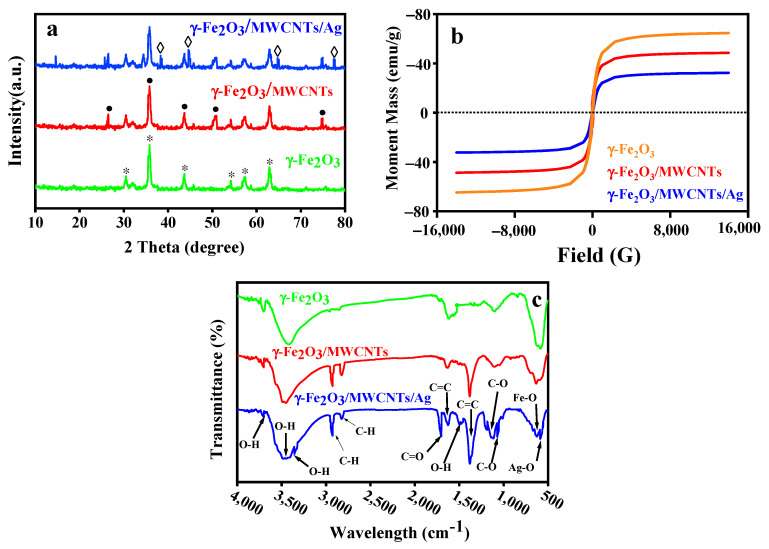
Crystallinity (**a**), magnetic (**b**), and chemical (**c**) properties of the γ-Fe_2_O_3_/MWCNT/Ag NC particles compared to those of γ-Fe_2_O_3_ NP and γ-Fe_2_O_3_/MWCNT.

The crystalline structure of γ-Fe_2_O_3_ NPs, γ-Fe_2_O_3_/MWCNTs, and γ-Fe_2_O_3_/MWCNTs/Ag NC particles was monitored by performing XRD, as shown in Figure 4a. The characteristic peaks appearing at 2θ of 63.3°, 57.6°, 54.4°, 43.8°, 35.5°, and 30.4°, are attributed to (440), (511), (422), (400), (311), and (220) crystalline planes of γ-Fe_2_O_3_, respectively (JCPDS No. 19–629 [39]). The sharp, narrow diffraction peaks seen at 2θ of 26.5°, 35.5°, 50.9°, 63.3°, and 74.7°, correspond to (002), (311), (102), (440) and (204) crystalline planes of MWCNTs, respectively (JCPDS No. 01-0646). After the addition of Ag NPs, new diffraction peaks emerged at 38.3°, 44.3°, 64.75°, and 77.4° which corresponded to Ag’s crystalline planes of (111), (200), (220), and (311), respectively (No. JCPDS NO 04-0783). These observations were similarly reported in [19,40].

The crystallite size of γ-Fe_2_O_3_ and Ag NPs was calculated via Debye-Scherrer’s Equation (10) [41]:

(10) $$D=\frac{0.98\lambda }{\beta COS\theta }$$

where *D* is the crystallite size, *λ* is the X-ray wavelength, *β* is the full width at half the maximum (FWHM), and *θ* is the Bragg angle. Accordingly, the crystallite size of γ-Fe_2_O_3_ and Ag NPs were calculated to be ~28.5 and 19.3 nm, respectively.

### 3.3. Magnetic Properties of the γ-Fe_2_O_3_/MWCNT/Ag NC Particles

The saturation magnetization (Ms) of γ-Fe_2_O_3_ NPs, γ-Fe_2_O_3_/MWCNTs, and γ-Fe_2_O_3_/MWCNTs/Ag NC particles was measured as 67.35, 52.34, and 38.21 emu g^−1^, respectively (Figure 4b). The reason for the loss of Ms in γ-Fe_2_O_3_/MWCNTs and γ-Fe_2_O_3_/MWCNTs/Ag NC particles compared to γ-Fe_2_O_3_ NPs could be the likely formation of an imprinting surface layer that enlarges the particles, thus lowering their magnetic properties. This performance was similarly reported by Qu et al. [42], and Moazzen et al. [32]. Having superb magnetic properties, γ-Fe_2_O_3_/MWCNTs/Ag NC particles can be readily separated from aqueous suspensions and do not cause any secondary contamination.

### 3.4. Surface Chemistry of the γ-Fe_2_O_3_/MWCNT/Ag NC Particles

The FTIR spectra of γ-Fe_2_O_3_ NPs, γ-Fe_2_O_3_/MWCNTs, and γ-Fe_2_O_3_/MWCNTs/Ag NC particles are shown in Figure 4c. The characteristic peak appearing at 593 cm^−1^ is attributed to the tensile vibration of Fe-O-Fe in γ-Fe_2_O_3_ [43]. The two characteristic peaks seen at 3580 and 3700 cm^−1^ represent the O–H group. The peak at 1610 cm^−1^ is assigned to the C=C tensile vibration of MWCNT. Moreover, the characteristic peaks emerging at 2926 cm^−1^ and 2854 cm^−1^ represent the asymmetric and symmetric tensile vibrations of C-H in the methylene group (–CH_2_) of MWCNTs, respectively. These arise from the imperfections of the graphitic structure generated during the preparation and subsequent coating of MWCNTs with the NPs [44]. The peak seen at 591 cm^−1^ in the γ-Fe_2_O_3_/MWCNTs/Ag NC spectrum confirms the formation of Ag-O on the NC surface [45]. In addition, the characteristic peaks appearing at 1038 cm^−1^, 1456 cm^−1^, 1700 cm^−1^, and 3350 cm^−1^ are attributed to C–O, OH, C=O, and OH groups of the NC particles, respectively [46]. 

**Figure 5:**
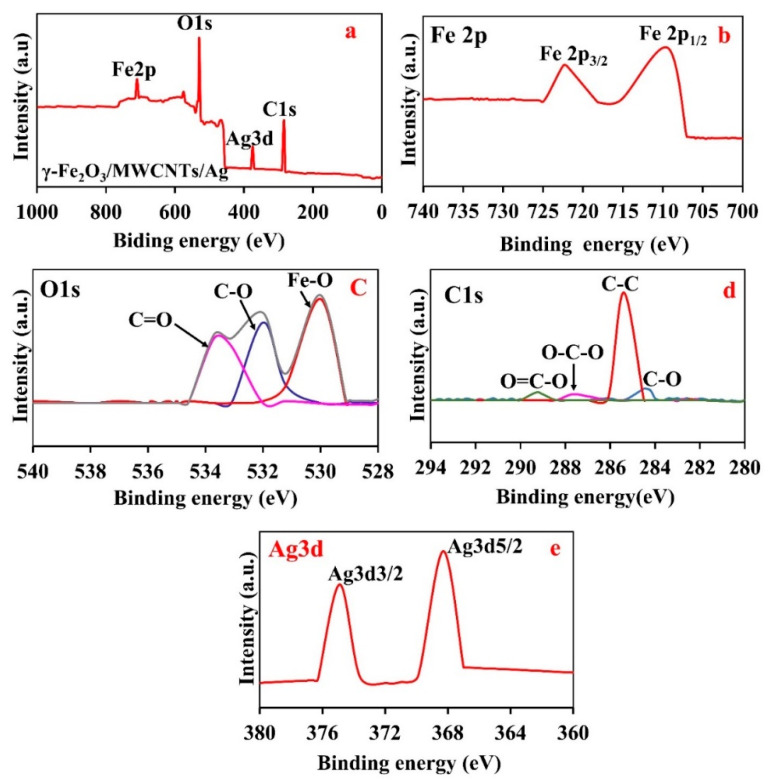
XPS analysis of the γ-Fe_2_O_3_/MWCNTs/Ag NC particles: (**a**) general XPS, (**b**) Fe2p, (**c**) O1s, (**d**) C1s, and (**e**) Ag3d spectrum.

Figure 5a demonstrates the general XPS spectrum of γ-Fe_2_O_3_/MWCNTs/Ag NC particles with the characteristic peaks of C, O, Fe, and Ag. The Fe 2p XPS spectrum (Figure 5b) contains two distinct peaks at 710.8 eV and 724.3 eV, corresponding to the typical Fe^3+^ binding energy of Fe 2p _3/2_ and Fe 2p _1/2_ due to the spin splitting of the 2p orbital electrons and indicates the presence of Fe in the form of Fe^+3^ [17,47]. Figure 5c shows the O1s XPS spectrum including one characteristic peak at 530 eV arising from γ-Fe_2_O_3_ and two peaks at 533.7 eV and 532 eV which correspond to C = O and C-O groups, respectively. These oxygen bearing groups originate from the defects of MWCNTs caused by nitric acid induced chemical oxidation [48]. The location of the peaks verifies the existence of the oxygen bearing functional groups on both ends and the sidewall of MWCNTs [49]. The C1s XPS peaks seen at 284.2 eV, 285.4 eV, and 287.3 eV (and 289.1 eV) represent C–C, graphitic carbon, and carboxyl group of MWCNTs, respectively (Figure 5d) [12,50]. The Ag3d XPS spectrum contains two distinct peaks at the binding energies of 374.1 eV and 368.3 eV, attributed to Ag3d_3/2_and Ag3d_5/2_, respectively (Figure 5e) [51]. The co-existence of numerous oxygen-containing groups on the surface of the purified MWCNTs can increase the number of carbon-oxygen bonds in the γ-Fe_2_O_3_/MWCNTs/Ag. The oxygen concentration increases with the refinement time using strong oxidants, as reflected in the increased intensity of the O1s peak, due to the emergence of new carbon-oxygen groups on γ-Fe_2_O_3_/MWCNTs/Ag NC particles. The reason behind the enhancement of conductivity after the acid treatment is the removal of the amorphous carbon which is largely susceptible to oxidation [51].

### 3.5. Specific Surface Area of the γ-Fe_2_O_3_/MWCNT/Ag NC Particles

The structural characteristics of the γ-Fe_2_O_3_/MWCNTs/Ag NC particles were studied using the BET analysis. The results of the N_2_ adsorption–desorption test are presented in Figure S1a,c,e for γ-Fe_2_O_3_ NPs, γ-Fe_2_O_3_/MWCNTs, and γ-Fe_2_O_3_/MWCNTs/Ag NC particles, respectively. The hysteresis loops emerging in the relative pressure range of 0.7 to 1.0 are consistent with a type IV isotherm according to the IUPAC classification and represent a mesoporous structure [52]. Furthermore, the Barrett–Joyner–Halenda (BJH) plots (Figure S1b,d,f) imply that the pore size distribution majorly lies in the mesoporous range (2–50 nm) [53]. The average pore size, specific surface area, and average pore volume of the NC particles are tabulated in Table S2. The specific surface area of γ-Fe_2_O_3_ NPs, Fe_2_O_3_/MWCNTs, and γ-Fe_2_O_3_/MWCNTs/Ag NC particles equals 41.96, 79.25, and 143.69 m^2^/g, respectively. The high specific surface area of the γ-Fe_2_O_3_/MWCNTs/Ag NC particles can be attributed to the attachment of Ag and magnetic NPs to MWCNTs, allowing for a greater adsorption of N_2_ molecules [32]. According to Table S2, the total pore volume and average pore size of the samples followed the order of γ-Fe_2_O_3_ > γ-Fe_2_O_3_/MWCNTs > γ-Fe_2_O_3_/MWCNTs/Ag NC. Therefore, while the specific surface area is promoted for the NC particles, the pore volume and pore size decline, most likely due to the attachment of the nanoparticles.

### 3.6. Thermal Properties of the γ-Fe_2_O_3_/MWCNT/Ag NC Particles

The TGA curves of γ-Fe_2_O_3_ NPs, γ-Fe_2_O_3_/MWCNTs, and γ-Fe_2_O_3_/MWCNTs/Ag NC particles are demonstrated in Figure S2. The γ-Fe_2_O_3_ NPs show a significant weight loss of 5.6% at 500 °C most likely due to the evaporation of the water adsorbed on the surface and due to the degradation of organic residues. No further weight loss is observed over 500 °C, implying the proper thermal stability of γ-Fe_2_O_3_ NPs at high temperatures [54]. However, as attached to MWCNTs, γ-Fe_2_O_3_ NPs demonstrated a reduced thermal stability, which is reflected in a weight loss of 81.4%. Such a weight loss might originate from the decomposition of some residual organic compounds or the oxidation loss of MWCNTs [38]. Additionally, it can be related to the catalytic role of the metallic oxide NC in the oxidation process of carbon [55]. The γ-Fe_2_O_3_/MWCNTs/Ag NC particles demonstrated a drastic weight loss (63.7%) at 500 °C, which was attributed to the removal of the covalently bonded organics [56].

### 3.7. Adsorption Behavior

#### 3.7.1. Taguchi Design of the Adsorption Experiment

**Figure 6:**
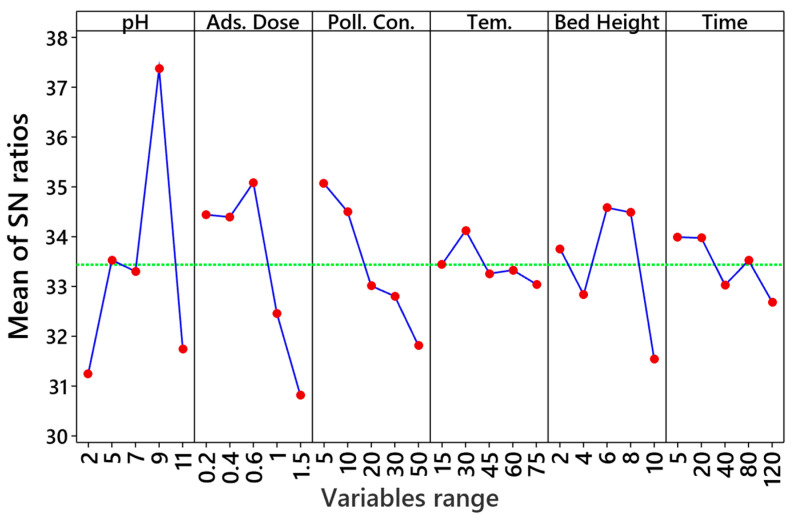
Signal-to-noise ratios of different experimental parameters of the SMT adsorption process (Ads: Adsorption, Poll. Con.: Pollution Concentration, Tem.: Temperature).

Table S1 presents the experimental design parameters as an L25 orthogonal array. According to this table, the SMT removal efficiency (RE) lies in the range of 15.74% to 90.97%. The optimum conditions to attain the highest RE of SMT include pH = 9, an adsorbent dosage of 0.6 g/L, SMT dosage of 5 mg/L, temperature of 30 °C, 6 cm bed height, and 5 min operation time. The difference between the RE predicted by the Taguchi model and the experimental RE was only 1.76, indicating the accuracy and validity of the design. Based on the computed signal to noise ratio (S/N), the order in terms of the contributing role of the evaluated factors in the final RE was as follows pH > adsorbent dosage > SMT concentration > bed height > time > temperature (Table S3 and Figure 6).

As shown in Figure 6, a remarkable SMT adsorption efficiency was achieved at higher S/N ratios. Among the operational parameters, pH shows the highest contribution to the adsorption process. Given that pH_pzc_ of the γ-Fe_2_O_3_/MWCNTs/Ag NC particles was 6, at the alkaline pHs, the surface of the γ-Fe_2_O_3_/MWCNTs/Ag NC particles was negatively charged and thus encouraged an electrostatic interaction with the cationic SMT molecules [57]. Adsorbent dosage follows pH in terms of importance in the adsorption process, as it is directly associated with the number of the accessible sites for adsorption [58]. An increase in the SMT initial concentration leads to a lower adsorption efficiency, which might originate from the saturation of the available adsorption sites with the SMT molecules [59]. The contact time also played a significant role at the beginning of the adsorption process, when a large number of active sites at the NC particle surface were available for SMT adsorption. As shown in Table S3, temperature had a less crucial contribution to SMT adsorption. High temperatures usually reduce the solution viscosity and thus provoke the mobility of the SMT cations [58].

#### 3.7.2. Adsorption Isotherms

**Figure 7:**
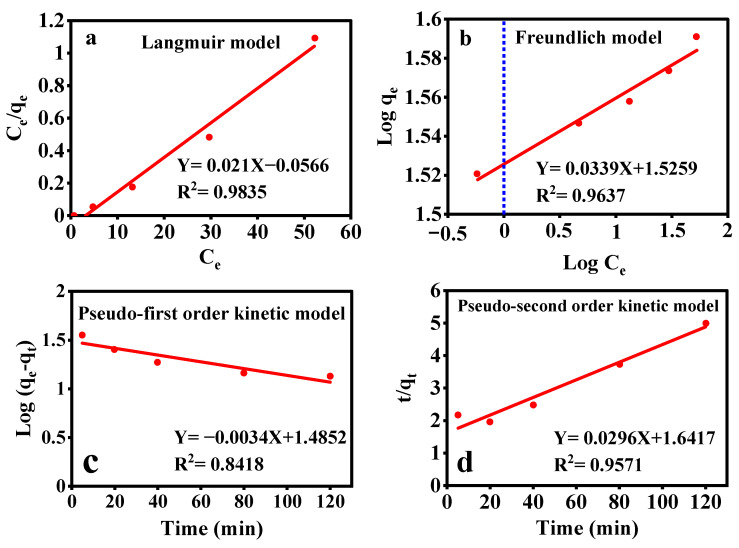
The equilibrium isotherms for adsorption of SMT on the γ-Fe_2_O_3_/MWCNTs/Ag NC particles: (**a**) Langmuir model, and (**b**) Freundlich model. Plots of pseudo-first (**c**) and pseudo-second order (**d**) kinetic models of the SMT adsorption on the γ-Fe_2_O_3_/ MWCNTs/Ag NC particles (experimental conditions included: 0.6 g/L of the nano-adsorbent, 10 mL of the SMT solutions (5–50 mg/L), and pH = 9).

The equilibrium isotherm is crucial to identify the affinity of the SMT molecules to the NC adsorbent [60]. To derive the adsorption isotherms, 0.6 g/L of the nano-adsorbent was dispersed in 10 mL of the SMT solutions (5–10 mg/L) at pH 9 and the suspension was kept for 120 min. The adsorption isotherms are shown in Figure 7a,b and Table S4. The highest adsorption capacity (*q_m_*) was measured to be 47.61 mg/g, which was significantly larger than that reported in the previous literature [61]. The Langmuir model indicates that the active sites are evenly distributed on the nano-adsorbent’s surface and the pollutant adsorbs in a monolayer on a homogeneous surface wherein the adsorption sites have equal affinity to the adsorbate. This nano-adsorbent has ionizable functional groups that act as suitable binding sites for SMT [62]. Differently, the Freundlich model is regarded as a multilayer adsorption mechanism that applies to heterogeneous surfaces. As seen in Table S4, the isotherm data were consistent with the Langmuir model (R^2^ = 0.9835). Additionally, 1/n was about 0.034 demonstrating that the adsorption process was appropriate under the experimental conditions [63].

#### 3.7.3. Adsorption Kinetics

The pseudo-first-order and pseudo-second-order models were used to determine the kinetic parameters of the adsorption process. The adsorption kinetics were monitored in a series of SMT aqueous solutions (10 mL) at pH 9 with the SMT initial concentrations of 5, 10, 20, 30, and 50 mg/L that were treated with the γ-Fe_2_O_3_/MWCNTs/Ag NC particles (0.6 g/L) for 5, 20, 40, 80, and 120 min. The kinetic behaviors, described through the pseudo-first and pseudo-second order models, are shown in Figure 7c,d, respectively, and the derived kinetic parameters are tabulated in Table S5. The *R^2^* values showed that the pseudo-second-order model (0.9571) was more suitable for describing the kinetic behavior of SMT adsorption. In addition, as deduced from the K_2_ values, the adsorption process fits well the pseudo-second-order kinetic model. Accordingly, the SMT adsorption process was driven chemically and is largely dependent on the presence of active sites on the nano-adsorbent surface [64].

#### 3.7.4. Adsorption Thermodynamics

**Figure 8:**
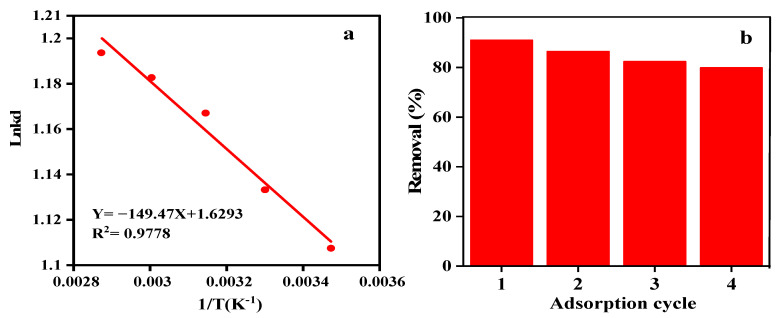
(**a**) The Van’t Hoff plot describing the thermodynamic behavior of SMT adsorption on the γ-Fe_2_O_3_/MWCNTs/Ag NC particles, and (**b**) reusability of γ-Fe_2_O_3_/MWCNTs/Ag NC adsorbent after 4 cycles.

Δ*S°* and Δ*H°* of the adsorption reactions can be derived from the slope and intersect of van’t Hoff plot (lnK_c_ vs. 1/T) (Figure 8a). As tabulated in Table S6, Δ*G°* is negative at different temperatures, indicating the spontaneous nature of the process. Furthermore, when the temperature rises, Δ*G°* declines. Therefore, the elevation of temperature can play a supportive role for the adsorption reaction. The magnitude and changes of Δ*G°* help comprehend whether the SMT adsorption is a physical or chemical process [65]. As deduced from the Δ*G°* measured in our study, the reaction between the SMT ions and the γ-Fe_2_O_3_/MWCNTs/Ag NC nano-adsorbent can be regarded a physical reaction. Additionally, the positive Δ*H°* implies the endothermic nature of the adsorption process. The positive Δ*S°* indicates that there is a considerable change in the system’s disarray over the course of the SMT adsorption cycle. Therefore, the collision of adsorbent and contaminant ions is consistent with the system’s irregularities [66].

#### 3.7.5. Adsorbent Reusability

Figure 8b shows that the adsorption capacity of the γ-Fe_2_O_3_/MWCNTs/Ag NC nano-adsorbent is maintained even after four adsorption/desorption cycles with a removal efficiency of ~80%. The slight reduction in the adsorption capacity is attributed to the protonation of the adsorption sites and occupation of the functional groups involved in the adsorption process. Secondly, the accumulation of SMT ions on the adsorbent’s surface restricts their further access to the binding sites [67]. It is thus believed that the γ-Fe_2_O_3_/MWCNTs/Ag NC nano-adsorbent offers a high adsorption capacity, reusability, and stability, thus holding potential for the effective and economical removal of pollutant ions from water solutions. 

#### 3.7.6. Antibacterial Activity

Table S7 presents the inhibition zone diameters caused by the γ-Fe_2_O_3_/MWCNTs/Ag NC particles in the presence of two bacterial strains, as compared with standard antibiotics. The NC particles were shown to offer a broad-spectrum activity with a consistent microbicidal efficiency against two tested bacteria. Both bacteria were affected by the NC particles. However, *S. aureus* bacteria responded more significantly compared to *E. Coli*, reflected in their larger inhibition zones of 17.8 ± 0.12 mm (versus 16.2 ± 0.1 mm for *E. Coli*). The possible antibacterial mechanism could be related to the entrapment of the bacteria by the quasi-aligned uniform long (>10 μm) nanotubes and destruction of their cell wall by the magnetic and Ag NPs [68]. The Ag and γ-Fe_2_O_3_ NPs on the MWCNTs contributed to improved antibacterial efficiency through further destruction of the cell membrane of the bacteria [69]. Olivi et al. [20] studied the antimicrobial activity of CNTs on the fungus *C. albicans* and reported that CNTs induce a profound antimicrobial effect. This performance can be associated with their physicochemical characteristics and the capability of the CNT networks in the entrapment of pathogens by van der Waals forces.

## 4. Conclusions

In the current study, we developed a highly efficient novel adsorbent made of γ-Fe_2_O_3_/MWCNTs/Ag NC using a facile and eco-friendly process. For the first time, such an NC material was synthesized using waste tires as the precursor of MWCNTs and the extract of *Viscum album* leaves as a reducing agent of Ag NPs. The Fe_2_O_3_/MWCNTs/Ag NC particles, as small as 75 to 100 nm in diameter, were used as nano-adsorbents for the removal of SMT from water. The optimum experimental conditions, according to the Taguchi design, were as follows: pH9, adsorbent dosage of 0.6 g/L, SMT dosage of 5 mg/L, temperature of 30 °C, 6 cm bed height, and a 5 min operation time. Under such conditions, the SMT removal efficiency could reach up to ~91%. The kinetic behaviour of the SMT adsorption matched the pseudo-second-order model well and the SMT adsorption process was driven chemically. From the thermodynamic point of view, the adsorption process was endothermic. The nano-adsorbent was reusable even after four successive adsorption cycles. In addition to reusability, which is an important parameter from a practical perspective, the sustainable production method of the NC particles holds great promise for further scalable development of this nanostructured, multi-functional nano-adsorbent in an eco-friendly manner.

## Data Availability

All data produced during this research project are openly available from the University of Birjand Research Data Archive.

## Supplementary Materials

The following supporting information can be downloaded at: https://www.mdpi.com/article/10.3390/nano12162798/s1, Figure S1. (a,c,e) BET isotherms for γ-Fe_2_O_3_, γ-Fe_2_O_3_/MWCNTs and γ-Fe_2_O_3_/MWCNTs/Ag; (b,d,f) pore size distribution of γ-Fe_2_O_3_, γ-Fe_2_O_3_/MWCNTs and γ-Fe_2_O_3_/MWCNTs/Ag, Figure S2: TGA curves of nanoadsorbents, Table S1: Controllable factors and their levels, experimental design using L25 and their responses, and the optimal conditions of SMT removal, Table S2: BET analysis, average pore size, and total pore volume of the γ-Fe_2_O_3_/MWCNTs/Ag, Table S3: Response of signal-to-noise ratios for SMT removal, criterion: Larger is better, Table S4: Langmuir and Freundlich isotherm parameters for SMT adsorption on γ-Fe_2_O_3_/MWCNTs/Ag, Table S5: Kinetic parameters of SMT uptake by the Fe_2_O_3_/MWCNTs/Ag, Table S6: Thermodynamic model parameters for SMT adsorption by γ-Fe_2_O_3_/MWCNTs/Ag, Table S7: Average of inhibition zones diameters for samples in different bacteria strains with three replicates.
