# Supplementary material for: Green, Sustainable Synthesis of γ-Fe2O3/MWCNT/Ag Nano-Composites Using the Viscum album Leaf Extract and Waste Car Tire for Removal of Sulfamethazine and Bacteria from Wastewater Streams
Source: Nanomaterials (Basel). 2022 Aug 15;12(16):2798. doi: 10.3390/nano12162798 (PMC9412352; doi:10.3390/nano12162798)
Supplement: Supplementary file 1 [file nanomaterials-12-02798-s001.zip › nanomaterials-1863646-supplementary.pdf]

Supplementary Materials

Table S1. Controllable factors and their levels, experimental design using L25 and their responses, and the optimal conditions of SMT removal

**Factors**	**Level 1**	**Level 2**	**Level 3**	**Level 4**	**Level 5**
pH	2	5	7	9	11
[1]Ads. Dose, g/l	0.2	0.4	0.6	1	1.5
[2]SMT Con, mg/l	5	10	20	30	50
[3]Tem. °C	15	30	45	60	75
Bed Height, cm	2	4	6	8	10
Time, min.	5	20	40	80	120

			**Factors Levels**			**RE**	
**[4]Exp. runs**	**pH**	**Ads. Dose**	**SMT Con.**	**Tem.**	**Bed height**	**Time**	**Response**
1	2	0.2	5	15	2	5	54.63
2	2	0.4	10	30	4	20	49.41
3	2	0.6	20	45	6	40	44.73
4	2	1	30	60	8	80	34.07
5	2	1.5	50	75	10	120	15.74
6	5	0.2	10	45	8	120	61.00
7	5	0.4	20	60	10	5	42.64
8	5	0.6	30	75	2	20	56.18
9	5	1	50	15	4	40	31.29
10	5	1.5	5	30	6	80	52.76
11	7	0.2	20	75	4	80	44.42
12	7	0.4	30	15	6	120	50.17
13	7	0.6	50	30	8	5	60.29
14	7	1	5	45	10	20	41.70
15	7	1.5	10	60	2	40	37.69
16	9	0.2	30	30	10	40	63.90
17	9	0.4	50	45	2	80	70.10
18	9	0.6	5	60	4	120	90.97
19	9	1	10	75	6	5	86.62
20	9	1.5	20	15	8	20	62.43
21	11	0.2	50	60	6	20	43.05
22	11	0.4	5	75	8	40	53.48
23	11	0.6	10	15	10	80	42.83
24	11	1	20	30	2	120	33.81
25	11	1.5	30	45	4	5	25.84

The optimal conditions

Factors	pH	Ads. Dose	SMT Con.	Tem.	Bed height	Time	[5]Pre. RE	[6]S/N	[7]Exp. RE
Opt. run	9	0.6	5	30	6	5	100	43.03	98.24

[1]Adsorbent dose, [2]Sulfamethazine concentration, [3]Temperature, [4]Experimental, [5]Predicted Removal Efficiencies, [6]Signal-to-noise, [7]Experimental

Table S2. BET analysis, average pore size, and total pore volume of the γ-Fe_2O_3/MWCNTs/Ag

Nano-adsorbent	BET area (m^2/g)	Total pore volume (Cm^3/g)	Average pore size (nm)
γ-Fe_2O_3	41.96	0.253	12.76
γ-Fe_2O_3/MWCNTs	79.25	0.217	10.25
γ-Fe_2O_3/MWCNTs/Ag	143.69	0.184	8.34

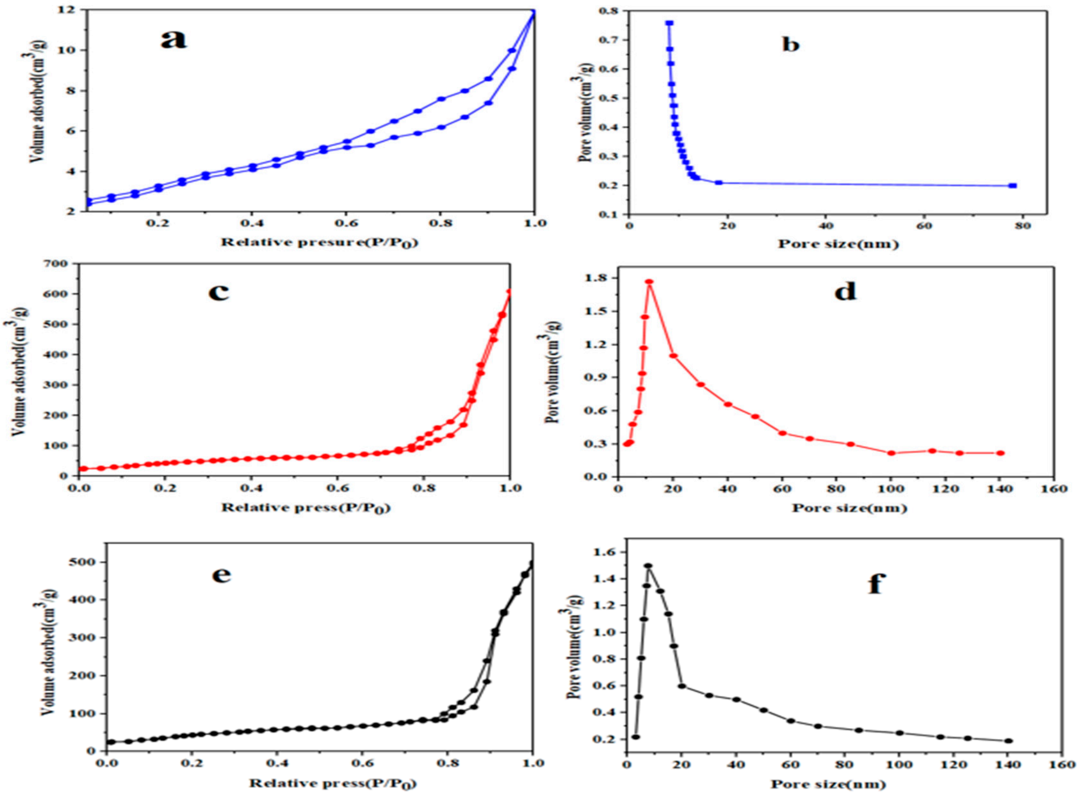

Figure S1. a, c, and e) BET isotherms for γ-Fe_2O_3, γ-Fe_2O_3/MWCNTs and γ-Fe_2O_3/MWCNTs/Ag; b, d, and f) pore size distribution of γ-Fe_2O_3, γ-Fe_2O_3/MWCNTs and γ-Fe_2O_3/MWCNTs/Ag.

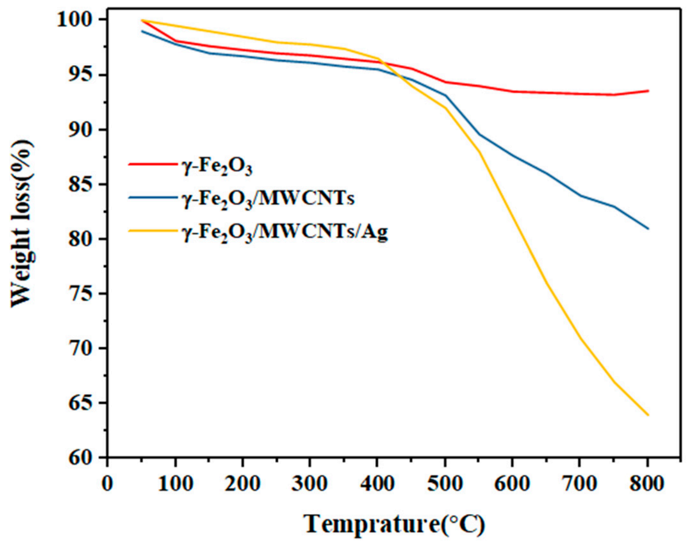

Figure S2. TGA curves of nanoadsorbents

Table S3. Response of signal-to-noise ratios for SMT removal, criterion: Larger is better

**S/N**						
**Level**	**pH**	**Ads. Dose**	**SMT. Con.**	**Time**	**Tem.**	**Bed height**
1	31.24	34.44	35.07	33.99	33.44	33.75
2	33.53	34.39	34.50	33.97	34.12	32.83
3	33.30	35.08	33.01	33.02	33.26	34.58
4	37.37	32.46	32.80	33.52	33.33	34.49
5	31.74	30.81	31.81	32.68	33.04	31.54
Delta	6.13	4.27	3.26	1.31	1.08	3.04
Rank	1	2	3	5	6	4

Table S4. Langmuir and Freundlich isotherm parameters for SMT adsorption on γFe_2O_3/MWCNTs/Ag

Langmuir isotherm			Freundlich isotherm		
**q_m(mg g^{-1})**	**K_L(L/mg)**	**R^2**	**n**	**K_f(mg/g)**	**R^2**
47.61	2.69	0.9835	29.4	33.3	0.9637

Table S5. Kinetic parameters of SMT uptake by the Fe_2O_3/MWCNTs/Ag

**Kinetic Models**	**Pseudo-first order**			**Pseudo-second order**		
**Parameters**	**q_e(mg/ g)**	**$k1$(min^{-1})**	**R^2**	**q_e(mg/g)**	**K_2(g/mg/ min)**	**R^2**
Values	30.56	0.0078	0.8418	37.17	4.40	0.9571

Table S6. Thermodynamic model parameters for SMT adsorption by γ-Fe_2O_3/MWCNTs/Ag

**T (K)**	**$Ln\ Kc$**	**ΔG°(J/mol)**	**ΔH°(kJ/mol)**	**ΔS°(kJ/mol)**
288	1.107	-2650.63	12.42	13.54
303	1.133	-2854.18		
318	1.167	-3085.37		
333	1.182	-3272.44		
348	1.194	-3454.56		

Table S7. Average of inhibition zones diameters for samples in different bacteria strains with three replicates.

Nanomaterials	Inhibition zones (mm)	
	E. coli	***S. aureus***
Zeocin	-	-
Kanamycin	12.4± 0.2	11.5 ± 0.3
γ- Fe_2O_3/MWCNTs/Ag (3%)	16.2 ± 0.1	17.8 ± 0.1
